# ASO Author Reflections: Current Treatment Options for Duodenal Adenocarcinoma—A Call for Collaborative Studies

**DOI:** 10.1245/s10434-018-6896-5

**Published:** 2018-10-16

**Authors:** Laura L. Meijer, Geert Kazemier

**Affiliations:** Department of Surgery, Cancer Center Amsterdam, Amsterdam UMC, VU University Amsterdam, Amsterdam, The Netherlands

## Past

The rarity of duodenal adenocarcinoma has led to a scarcity of studies performed to evaluate outcomes after varying treatment options. Similar to the vast majority of malignant solid tumor types, radical surgical resection remains the only curative treatment option, often performed by pancreaticoduodenectomy or segmental resection. However, the most optimal surgical technique is debated.^1^,^2^ During recent years, administration of adjuvant therapy also has been explored to enhance survival further for patients with duodenal adenocarcinoma.^3^ Most schedules are currently mimicking chemo(radio)therapy strategies used for patients with colorectal cancer. However, this rationale is based primarily on tumor location and phenotypic resemblance, whereas therapeutic choices based on randomized trials are absent. Practical guidelines for treating patients with duodenal adenocarcinoma are needed. Our study provided an overview of the current literature to illustrate the outcomes and different treatment options for patients with duodenal adenocarcinoma. This could help in future trial design and optimization of clinical care.

## Present

This systematic review and meta-analysis involved 26 articles for critical appraisal.^4^ Almost all the included studies were retrospective cohort studies. Of all 1685 evaluated patients, 71% underwent resection with curative intent, which resulted in a pooled 5-year survival rate of 46%. Interestingly, no significant difference in terms of survival was found between segmental resection and pancreaticoduodenectomy. This result justifies segmental resection when radical resection margins can be achieved with that technique. In the studies reviewed, administration of adjuvant therapy did not enhance survival outcomes. Some caution is warranted, however, because most of the studies did not correct for prognostic factors such as lymph node involvement, and treatment schedule details often were not reported. Of the specified schedules, fluorouracil-based chemotherapeutic regimens were most commonly used, alone or combined with a platinum-based compound. Adequate stratification based on prognostic factors and type of adjuvant therapy are both needed to conclude correctly on the role of adjuvant therapy for duodenal adenocarcinoma.

## Future

Combining efforts in international collaborations is pivotal to enablement of large-scale studies on the added value of (neo)adjuvant treatment for patients with duodenal adenocarcinoma. Due to scarcity of the tumor, however, even this might not be sufficient to unravel these pressing issues. Potentially, novel insights into tumor biology and genomic analysis would allow for a more fundamental approach to choosing systemic therapy regimens to optimize treatment for patients with duodenal adenocarcinoma. Moreover, studies also are needed to evaluate optimal palliative care because the current studies are even more rare and heterogeneous. Finally, local treatment of liver metastases and cytoreductive surgery with hyperthermic intraperitoneal chemotherapy are considered standard of care for patients with metastatic colon carcinoma. Both still are unexplored fields for patients with duodenal adenocarcinoma.^5^ These therapeutic options deserve to be considered and properly investigated, at least in cohort studies, to evaluate their true merits for patients with duodenal adenocarcinoma.
